# Chronic endometritis and altered embryo implantation: a unified pathophysiological theory from a literature systematic review

**DOI:** 10.1007/s10815-020-01955-8

**Published:** 2020-10-06

**Authors:** Giovanni Buzzaccarini, Amerigo Vitagliano, Alessandra Andrisani, Carla Mariaflavia Santarsiero, Rossana Cicinelli, Claudia Nardelli, Guido Ambrosini, Ettore Cicinelli

**Affiliations:** 1 Gynecological Clinic, UOS Medically Assisted Procreation, University of Padova, via Nicolò Giustiniani 3, Padova, Italy; 2 Second Unit of Obstetrics and Gynecology, Department of Biomedical Sciences and Human Oncology, University of Bari “A. Moro”, Piazza G. Cesare 11, Bari, Italy

**Keywords:** Chronic endometritis, Infertility, Pathophysiology, IVF, Embryo transfer, Recurrent pregnancy failures, Recurrent pregnancy loss

## Abstract

**Purpose:**

Chronic endometritis (CE) is a frequent hysteroscopic and histological finding which affects embryo transfer implantation during IVF-ICSI cycles. In particular, CE impairs proper decidualization and, subsequently, implantation. Although this correlation has been clearly clarified, a pathophysiological explanation assembling all the studies performed has not been elucidated yet. For this reason, we have structured a systematic review considering all the original articles that evaluated a pathological element involved in CE and implantation impairment.

**Methods:**

The authors searched electronic databases and, after screening, collected 15 original articles. These were fully scanned and used to create a summary pathway.

**Results:**

CE is primarily caused by infections, which lead to a specific cytokine and leukocyte pattern in order to prepare the uterus to fight the noxa. In particular, the immunosuppression requested for a proper semi-allogenic embryo transfer implantation is converted into an immunoreaction, which hampers correct embryo implantation. Moreover, endometrial vascularization is affected and both irregular vessel density and luminal thickening and thrombosis reduce what we have first identified as endometrial flow reserve. Finally, incorrect uterine wave propagation could affect embryo contact with decidua.

**Conclusion:**

This is the first summary of evidence on CE pathophysiology and its relationship with infertility. Understanding the CE pathophysiology could improve our knowledge in embryo transfer success.

## Introduction

Chronic endometritis (CE) is a persistent inflammatory disorder of the endometrial lining, characterized by superficial endometrial edematous change, high stromal cell density, dissociated maturation between epithelium and stroma, and infiltration of endometrial stromal plasmacytes (ESPCs) [1–5]. The pathogenesis of CE seems to be related to a qualitative and quantitative alteration of endometrial microbioma, with the abnormal proliferation of different types of microorganisms, mainly gram-negative and intracellular bacteria (i.e., *Enterococcus faecalis*, *Mycoplasma*, *Ureaplasma*, *Chlamydia*, *Escherichia coli*, and *Streptococcus* spp.) [2, 6–9]. As proof of the infectious etiology of CE, several studies have found that specific antibiotic cycles can cure CE in the majority of patients [1, 7, 10].

In most cases, women with CE are asymptomatic or display mild disturbances, such as abnormal uterine bleeding (AUB), dyspareunia, pelvic discomfort, and leukorrhea [11–13]. Moreover, CE cannot be identified by ultrasound examination due to a lack of specific ultrasound markers [8]. For these reasons, CE is often overlooked or diagnosed incidentally during the diagnostic workup of different gynecological disorders including AUB, infertility, or chronic pelvic pain [14].

Fluid hysteroscopy plays a central role in the diagnostic challenge of CE. This technique allows the identification of some endometrial modifications that are specific for CE (i.e., focal or diffuse micropolyps, stromal edema, focal hyperemia, strawberry aspect, and endometrial hemorrhagic spots), as recently demonstrated by our group [15].

The current gold standard for CE diagnosis is endometrial biopsy with histological analysis, where the detection of plasma cells within endometrial stroma is the main diagnostic marker [7]. A plasma cell is a type of white blood cell which is derived from B lymphocytes; it is capable of secreting immunoglobulins and is the main cell responsible for humoral immunity. Different studies have shown that traditional staining with hematoxylin and eosin (H&E) may be not sufficiently accurate for highlighting endometrial plasma cells due to their morphological similarities with fibroblasts. Differently, the immunohistochemical staining for CD138 was associated with lower intra- and interobserver variability between pathologists in the detection of plasma cells and has now become the reference standard technique for diagnosing CE [16–18].

During the last several decades, CE has attracted a great deal of attention among scientists and fertility care providers due to its potential association with reproductive issues. In particular, several studies have found that CE is highly prevalent among women suffering from unexplained infertility (from 40.7 to 55.7%), recurrent IVF failures (from 13.95 to 57.55%), and repeated early pregnancy loss (from 42.9 to 56%). Importantly, adequate therapy of CE can lead to a complete normalization of endometrial histology and to the restoration of the reproductive function in women with CE [19–24].

In recent years, many authors have investigated the possible mechanisms by which CE may hamper the reproductive health of the endometrium, but the pathophysiological pathway has not been fully assessed yet. In this present study, we aimed to provide the first summary of evidence on the pathophysiological mechanisms involved in CE-related reproductive impairment.

## Materials and methods

### Study design

This is a systematic literature review on the pathophysiological mechanisms involved in CE-related reproductive issues. As it was a review of published data, institutional review board approval was not required.

### Search strategy

Electronic databases (ScienceDirect, MEDLINE, Scopus, Embase, the Cochrane Library, Clinicaltrials.gov, EU Clinical Trials Register, and the World Health Organization International Clinical Trials Registry) were searched for articles indexed from the inception to September 2019. The search was conducted adopting the following keywords: “chronic endometritis AND (infertility OR fertile OR fertility OR miscarriage OR implantation failure OR implantation OR endometrial receptivity OR decidualization OR ART OR IVF OR mechanisms OR causes OR pathway OR pathophysiology).” Furthermore, the reference list of all identified articles and reviews were accurately examined to avoid any missing data.

### Inclusion criteria

All the studies assessing the pathophysiology of infertility in women with CE were evaluated. Chronic endometritis was defined as a chronic inflammation of endometrium, diagnosed by the histologic presence of one or more plasma cells in the endometrial stroma in the entire section. Articles evaluating other types of endometrial inflammation (such as acute, subacute, or tubercular endometritis) were not included.

No restrictions on the year of publication were applied. The search and the selection criteria were restricted to English language and only studies with full text available were considered suitable for inclusion.

We included only original articles both on humans and animals. However, we did not find any animal study which matched our inclusion criteria. Reviews, systematic reviews and meta-analysis were excluded. The studies evaluated were primarily case-control studies, cohort studies, and retrospective studies.

### Study selection and data extraction

The electronic and reference list searches were performed independently by G.B., R.C., and C.M.S. These investigators then screened titles and abstracts. The results were then compared and any disagreement was resolved by discussion with other reviewers (E.C. and A.V.).

### Included studies

The electronic search identified 419 records. Forty-one full-text articles were evaluated, of which 26 were excluded. A total of 15 manuscripts were considered eligible for inclusion in the study. Table 1 shows the main characteristics of the studies considered.

**Table 1:** Main characteristics of the studies enrolled

Authors and years (reference)	Study design, country, and time of realization	Participants and main inclusion criteria	Samples, timing, and methods	Main outcomes
Cicinelli, 2008	Prospective controlled study, Italy, from January 2005 to April 2006	2190 women undergoing hysteroscopy for different indications. Vaginal and endometrial samples were collected from 438 women with a CE diagnosis at hysteroscopy and 100 women with no signs of CE (controls).	Diagnostic office hysteroscopy in the follicular phase of the menstrual cycle. Women had a vaginal swab taken and an endometrial sampling using a 3-mm Novak’s curette connected to a 20-mL syringe. Cultures for common bacteria, *Neisseria gonorrhoeae* and *Mycoplasma* and molecular biology testing for *Chlamydia* were performed.	More than 70% of CE cases resulted from non-gonococcal and non-chlamydial infections. Common bacteria and Mycoplasma were the most frequent etiologic agents. Vaginal cultures have low concordance with endometrial cultures.
Mishra, 2008	Retrospective case-control study, India, from January 2005 to June 2007	20 granulomatous endometritis, 10 chronic non-specific endometritis, and 30 controls	Endometrial curettings were obtained in the fourth week of the menstrual cycle. Immunohistochemistry for ER, PR, and Ki-67.	Higher ER, PR and Ki-67 expression in endometrial glandular and stromal cells. Endometrial inflammation interferes with local expression of ER, PR, and Ki-67.
Cicinelli, 2009	Prospective controlled study, Italy, from January 2005 to April 2006	181 women in whom diagnostic hysteroscopy had showed the presence of CE.	Mini-hysteroscopy in the follicular phase of the menstrual cycle. Vaginal and cervical swabs were obtained and endometrial sampling took place, using a 3-mm Novak curette connected to a 20-ml syringe. Histological examination, cultures for common bacteria, *Neisseria gonorrhoeae* and *Mycoplasma* and molecular biology testing for *Chlamydia* were performed.	Both vaginal and endocervical cultures have low concordance with endometrial cultures in women with diagnosed CE.
Matteo, 2009	Case-control study, Italy, no dates reported	23 infertile women. 9 with CE diagnosed and 14 without.	Diagnostic office mini-hysteroscopy and endometrial biopsy in the follicular phase of menstrual cycle. All patients, in the late secretory phase (LS) of the subsequent spontaneous menstrual cycle, underwent endometrial biopsies by a 3-mm Novak’s curette connected to a 20 mL syringe. Histological examination, flow cytometry, and antibody labeling.	The secretory endometrium of patients with CE displayed significantly lower percentage of CD56+ CD16- and of CD56bright CD16- cells as compared with group CE-, while the percentage of CD3+ cells was significantly higher.
Kitaya, 2010	Case-control study, Japan, no dates reported	76 infertile women with histological biopsy. 22 of them diagnosed with CE	Endometrial specimens were obtained from patients with unexplained infertility and who had undergone biopsy in search of endometrial pathology on days 6 to 8 after urinary luteinizing hormone-surge detection. Histological Examination and Immunoassay were performed.	B cell density is higher in CE endometrium. CXCL13 expression in CE microvascular endothelial cells is higher.
Carvalho, 2013	Observational cohort study, Brazil, from 2009 to 2010	435 infertile women	Diagnostic hysteroscopy followed by blind endometrial aspiration biopsy using a silicone urethral catheter number 8. All the biopsies were performed after the 10th day of the menstrual cycle until the 5th postovulatory day.	Association between vascular changes, CE, and infertility.
Di Pietro, 2013	Case-control study, Italy, no dates referred	16 women with hysteroscopic and histological diagnosis of CE and 10 healthy women as controls.	Hysteroscopy and endometrial sampling using a Novak’s curette connected to a 20-mL syringe. The procedures were performed in the secretory phase during the implantation window. Histological examination and gene Expression Profiling by Real-Time RT-PCR.	IGFBP1, BCL2, and BAX are upregulated, while IL11, CCL4, IGF1, and CASP8 are downregulated.
Kitaya, 2014	Case-control study, Japan, From January 2011 to December 2012	179 infertile women with repeated implantation failure (RIF). 59 were diagnosed with CE.	Fluid hysteroscopy was performed on days 6–12 of the menstrual cycle. Histological biopsy was performed using a 3-mm-wide curette. Histological Examination and Immunoassay were performed.	The density of IgM+, IgA1+, IgA2+, IgG1+, and IgG2+ stromal cells were significantly higher in the RIF-CE group than that in the RIF-non-CE and control group.
Pinto, 2015	Case-control study, Italy, from March 2012 to December 2013	45 women referred for hysteroscopy with diagnosis of CE. 45 age-matched women as controls with no evidence of CE at hysteroscopy and biopsy.	Hysteroscopy, histology examination, and TVS evaluation of the EW pattern during the periovulatory (days 11–14) and midluteal (days 19–22) phases of the same cycle.	CE could influence uterine contractility. CE induces a reduction of retrograde motility in the periovulatory phase and an increase of anterograde and retrograde motility in the midluteal phase.
Kushnir, 2016	Retrospective cohort study, USA, from January 2014 to August 2015	55 patients with recurrent pregnancy loss (RPL) and/or implantation failure (RIF).	Hysteroscopic and histological examination. Serum examination.	No findings in the periphery serum that support the hypothesis that CE may, at least in some cases, have an autoimmune component. Dysregulation of local inflammatory pathways may play a role in the pathophysiology of RPL as well as RIF.
Wu, 2017	Case-control study, Japan, no dates reported.	17 patients, 9 CE (5 endometriosis), 8 non-CE (4endometriosis)	Hysteroscopy and curettage performed at 7 or 8 days after predicted ovulation. Endometrial Cultures, Immunoassay, Gene Expression Profiling by Real-Time RT-PCR, Immunohistochemistry	Increased cell numbers and reduced secretion of PRL and IGFBP-1. Increased expressions of ERα, ERβ, PRA, and PRB.
Moreno, 2018	Case-control study, Italy, no dates reported	113 women with CE diagnosed using endometrial histology, hysteroscopy, and/or microbial culture.	Hysteroscopy performed in the follicular phase (cycle day 7–12) and endometrial sampling using a 3-mm Novak curette connected to a 20-mL syringe. Microbiological culture and molecular microbiology diagnosis by RT-PCR.	RT-PCR effectively detects and quantifies bacterial DNA from chronic endometritis-causing pathogens in endometrial samples providing a feasible, faster, and cheaper method for the diagnosis of chronic endometritis.
Di Pietro, 2018	Case-control study, Italy, from October 2016 to March 2017	15 women with hysteroscopic and histological diagnosis of CE and 15 healthy women.	Hysteroscopy was performed in follicular phase. Endometrial biopsies were taken with Pipelle de Cornier. Histological Examination and Serum Examination, Gene Expression Profiling by Real-Time RT-PCR.	Upregulation of miR-27a-3p and miR-124-3p in the endometrium and serum from women with CE and an anti-correlation relationship between miR-27a-3p and IGF1 in endometrium.
Liu, 2019	Case-control observational study, China	130 infertile women. 12 with CE diagnosed.	Endometrial fluid and scratch collection 7 days after LH surge. Endometrial scratch was performed with a Pipelle (Prodimed). Genomic DNA Extraction and PCR amplification using primers targeting the 16S rRNA gene.	Defining endometrial microbiota of women with or without CE.
Wang, 2019.	Case-control study, China, from February 2015 to July 2018	75 CE women with recurrent implantation failure and 75 women with male factor infertility.	Office hysteroscopy was scheduled during the follicular phase (between cycle day 8 to 12) of the menstrual cycle. All women with clinical CE by hysteroscope underwent endometrial biopsy using a curette for histological confirmation. Subsequently, gene expression profiling by real-time RT-PCR, immunohistochemistry, and immunoassay.	Decreased endometrial TGF-β and IL-10 expression and increased IL-17 expression. Increased autophagy (LC3-II) and mTORC1 downregulation.

Fifteen studies performed hysteroscopy (Carvalho 2013 Cicinelli 2008, Cicinelli 2009, Di Pietro 2013 Di Pietro 2018, Kitaya 2010, Kitaya 2014, Kushnir 2016, Liu 2019, Matteo 2009, Mishra 2008, Moreno 2018, Pinto 2015, Wang 2019, Wu 2017). One study performed vaginal swab (Cicinelli 2008). Nine studies performed biopsy via Novak curettage (Cicinelli 2008, Cicinelli 2009, Di Pietro 2013, Kitaya 2014, Matteo 2009, Mishra 2008, Moreno 2018, Wang 2008, Wu 2017). On the other hand, 2 studies performed biopsy via Pipelle (Di Pietro 2018, Liu 2019). One study performed blind endometrial biopsy (Carvalho 2013).

The time of intervention was different. Carvalho (2013) performed biopsy between 10 and 5 days post ovulation. Cicinelli (2008, 2009), Di Pietro (2018), Matteo (2009), and Moreno (2018) carried out the biopsy during the follicular phase. By contrast, Di Pietro (2013) and Mishra (2008) performed the intervention in the secretive phase. Liu (2019) administered biopsy on day 7 from luteinizing hormone (LH) surge. Kitaya (2010) performed biopsy from days 6 to 8 post LH surge. Kitaya (2014) conducted the procedure from days 6 to 12 post period, and Wang (2019) from day 8 to 12 post period. Wu (2017) from days 7 to 8 after predicted ovulation. Finally, Matteo (2009) performed biopsy on the late secretive phase.

Moreover, in order to perform a microbiological study, 4 studies used cultures (Cicinelli 2008, Cicinelli 2009, Moreno 2018 and Wu 2017).

Only one study performed vaginal ultrasound evaluation (Pinto 2015).

Gene expression profiling RT-PCR was used in 8 studies (Cicinelli 2008, Cicinelli 2009, Di Pietro 2013, Di Pietro 2018, Liu 2019, Moreno 2018, Wang 2019, Wu 2017). Three studies used immunohistochemistry (Mishra 2008, Wang 2019, Wu 2017). Three studies used immunoassay (Kitaya 2014, Wang 2019, Wu 2017). One study examined serum (Kushnir 2016) and another study performed antibody labeling and flow cytometry (Matteo 2009).

## Results and discussion

Chronic endometritis (CE) is a disease characterized by one main feature: inflammation. Actually, while the biopsy findings are defined and classified, the pathophysiological pathway is still unclear. Moreover, the relationship between CE and infertility or repeated implantation failure is still under investigation.

Firstly, it must be considered that implantation is the result of a complex interaction between the blastocyst and the endometrium. Different signaling pathways participate in this unique biological link and an appropriate endometrium is requested for the implantation success. This unstable balance can easily be altered by embryonic factors (and this is not our study concern) or endometrial factors. In this case, CE is what inhibits the endometrial ability to achieve a successful implantation.

Our pathophysiological model starts with the following consideration: CE inflammation is mainly caused by infection. Different microorganisms have been detected and various antibiotic protocols have enhanced IVF success after administration in CE infertile women. However, we are of the opinion that the infection is only a trigger of a more complex sequence that consists of an academically organized flowchart.

An altered cytokine and chemokine secretion induces altered leukocyte population recruitment. These two conditions impact on uterine contractility, endometrial function in decidualization, and receptivity and vascularization. The main role is played by autophagy, which is necessary for achieving implantation.

### Infection and autoimmunity

In the majority of patients with CE, microbiological analyses (cultures or RT-PCR analyses) are positive for endometrial microorganisms. However, there are also specific cases in which we are unable to identify any microorganisms, or the microorganism is not culturable, raising the doubt of a possible autoimmune pathogenesis of CE. It is to be noted that CE is characterized by plasma cell infiltrates, which are associated with practically all organ autoimmune responses, including rare autoimmune diseases of the reproductive system (i.e., autoimmune oophoritis). To test the hypothesis of CE as an autoimmune condition, a recent study (Kushnir et al. 2016) [25] compared different inflammatory and autoimmune markers between infertile women with CE versus infertile women without CE. The authors failed to demonstrate different values of total immunoglobulins, antinuclear antibodies, thyroid antibodies, and antiphospholipid antibodies between the two groups under comparison (*p* > 0.05), drawing the conclusion that CE does not have a substantial autoimmune component. Although these results need further confirmation, the hypothesis of an autoimmune-driven CE cannot be sustained at present. Accordingly, our model starts considering infections as the main immune trigger for CE.

As is already known, the uterine cavity is not sterile in physiological conditions, but is inhabited by a plethora of microorganisms mainly belonging to *Lactobacilli* species. Therefore, the isolation of endometrial microorganisms does not necessarily correlate with endometrial inflammation [26]. Cicinelli et al. investigated CE microorganisms by performing both hysteroscopic and histologic exams, in addition to endometrial and vaginal cultures [2, 9]. The findings can be summarized as follows:

1. CE endometrium showed a prevalence of common bacteria. In particular, streptococci were found in 27.9% of cases and bacteria from intestinal flora (*Enterococcus faecalis* and *Escherichia coli*) were detected in 25.5% of cases. *Ureaplasma urealyticum* was detected in 10.0%, and *Chlamydia* in only 2.7% of cases. No cases of *N. gonorrhoeae* were found.
2. Endometrial, vaginal, and endocervical cultures in CE women were compared to investigate the percentage of concordance with the etiologic agent. There was a statistically significant difference for *Streptococcus*, *Staphylococcus*, *E. faecalis*, and *U*. *urealyticum* (which were found to be more prevalent in vaginal than in endometrial samples).
3. Both vaginal and endocervical cultures have low concordance with endometrial cultures. In particular, positive *Staphylococcus* endometrial cultures did not have any positive vaginal findings. On the other hand, *Chlamydia* has a 100% concordance between endometrial culture and endocervical culture. By contrast, in the majority of cases which proved positive for *Ureaplasma* and yeast, the microorganisms were also detected at the vaginal level.

However, bacteriological cultures are limited to those microorganisms that are able to grow under traditional culture. About 10% of cultures were negative in the endometrium suffering from CE (with histological diagnosis), and this was ascribable to the presence of other microorganisms (viruses or non-culturable bacteria) or the presence of autoimmune factors.

Moreno (2018) reported other results. In particular, streptococci were the most abundant bacteria detected (47%), followed by enterococci (15%), *E. coli* (12%), *K. pneumoniae* (5%), staphylococci (3%), and *Mycoplasma hominis* (2%) [8]. These results are quite similar to previous findings and, specifically, it is generally accepted that streptococci are the most prevalent microorganism in CE endometrium. Interestingly, *G. vaginalis* was detected in 7% of the samples analyzed and this is in line with Liu 2019, who found an increase in CE endometrium [27]. On the other hand, *C. trachomatis* and *N. gonorrhoeae* were undetectable in all tested samples, which is in agreement with the limited role of sexually transmitted disease in the CE pathogenesis of previous studies [8].

Liu (2019) studied CE microbioma on the 7th day after the LH peak (midluteal phase), the most favorable period for possible implantation, with PCR [27]. The main findings are that CE endometrial cavity shows a lower percentage of *Lactobacillus* species which are negatively correlated with *Gardnerella*, *Anaerococcus*, *Finegoldia*, and *Staphylococcus*. These are reported to be associated with preterm delivery and bacterial vaginosis. *Lactobacillus* species are, indeed, known to inhibit other bacteria by producing hydrogen peroxide and lactic acid. For this reason, *Lactobacillus* plays a major role as protective bacteria against dangerous microorganisms [28]. Table 2 summarizes the percentage of all the different species detected in these 4 studies [2, 8, 9, 27].

**Table 2:** microorganism percentage detected at CE biopsies

Authors and years	Analysis method	Microorganisms detected in CE endometrium
Cicinelli 2008	Hysteroscopic and histologic exam, microorganism cultures.	Streptococci 27,9% Intestinal flora (*Enterococcus faecalis* and *Escherichia coli*) 2.5% *Ureaplasma urealyticum* 10% *Chlamydia* 2.5% Staphylococci *Neisseria gonorrhoeae* 0%
Cicinelli 2009	Microorganism cultures, PCR	Streptococci 28,7% Intestinal flora (*Escherichia coli*, *Enterococcus faecalis*) 26,6% *Ureaplasma urealyticum* 11% *Chlamydia trachomatis* 2,8% Staphylococci 4%
Moreno 2018	PCR	Streptococci 47% *Enterococcus faecalis* 15% *Escherichia coli* 12% *Gardnerella vaginalis* 7% Staphylococci 3% *Mycoplasma hominis* 2% *Chlamydia* 0% *Neisseria gonorrhoeae* 0%
Liu 2019	PCR	(vs non-CE endometrium) Lactobacilli ↓ *Atopobium* ↑ *Bifidobacterium* ↑ *Gardnerella* ↑ *Prevotella* ↑ *Stenotrophomonas* ↑ Others ↑

In conclusion, both an increase in dangerous microorganisms and a decrease in protective microorganisms can lead to potential uterine damage. The link between infections and the development of inflammation is a clear pathway step, since a vast number of studies have clarified that organ inflammation diseases can be caused by microorganisms. To clarify, HAV, HBV, and HCV are well-known causes of chronic hepatitis and this can lead both to cirrhosis and liver hepatocarcinoma. On the other hand, Alzheimer’s disease is now also related to viral infections such as *Human herpesvirus 1* (HHV-1), *Cytomegalovirus* (CMV), and *Human herpesvirus 2* (HHV-2) [29]. For this reason, it seems reasonable that an organ which is more prone to extra-corporeal contamination can also develop a chronic inflammatory disease. In particular, the link between infections and inflammation is the first step in our pathway and it is mediated by an interaction between inflammation stimuli such as microbial products, interleukin-1β (IL-1β), interleukin-6 (IL-6) and tumor necrosis factor-α (TNF-α), and toll-like receptors (TLRs), IL-1 receptor (IL-1R), IL-6 receptor (IL-6R), and the TNF receptor (TNFR) [30]. Lipopolysaccharide, in particular, is the major component of the outer membrane of gram-negative bacteria and the ligand to toll-like receptor 4 expressed on their host cells. For this reason, lipopolysaccharide derived from gram-negative bacteria may be a trigger and mediator of chronic endometritis.

### Cytokine dysregulation

Cytokines are inflammation mediators. Infection causes an aberrant local microenvironment due to an altered secretion of paracrine factors. Moreover, the endometrial microvascular endothelium plays a critical role by mediating the recruitment of leukocytes. In particular, the following signaling pathways have been reported in association with CE:

1. CE downregulates IL-11 expression. A possible explanation can be found in miR-124-3p upregulation, which is a negative modulator of IL-11 [31, 32]. To clarify, IL-11 activity, binding to IL-11R alpha, helps trophoblast invasion by inducing endometrial epithelial adhesion molecule mRNA expression. Moreover, IL-11 plays a key role in decidualization, since it progresses progesterone-induced decidualization of human endometrial stromal cells, and in endometrial vascularization, promoting angiogenesis and/or remodeling of the maternal vasculature [33].
2. CE downregulates CCL-4. CCL-4 is a chemokine that attracts natural killer (NK) cells and macrophages, whose activity is important for implantation, as they produce angiogenic factors, such as vascular endothelial growth factor (VEGF) promoting spiral artery remodeling which supports the implantation of the trophoblast. Specifically, CCL-4 can be responsible for uterine NK recruitment from plasma NK cells [31].
3. CE upregulates IGFBP-1, whose expression is normally increased during decidualization [31]. However, Wu et al. (2017) found a discordant result regarding IGFBP-1 [34], which is downregulated in CE. In addition to this, it must be considered that IGFBP-1 is a modulator of trophoblast invasion, and specifically a negative modulator [35]. Probably, IGFBP-1 is a regulator of decidualization, acting as a damper of this complex interaction between embryo and decidua.
4. CE downregulates IGF-1, which mediates the effects of estrogen on endometrial proliferation during the proliferative phase of the endometrial cycle. An explanation can be found on the role of miR-27a-3p, which is a negative modulator of IGF-1 and is upregulated in CE. A significant anti-correlation ratio was found for miR-27a-3p and IGF-1 [31, 32].
5. CE upregulates both BCL-2 and BAX. BCL-2 inhibits CASP-8, which is a pro-apoptotic gene and has a role in endometrial decidualization. The BCL-2 overexpression in women with CE makes the endometrial cells more resistant to apoptosis. However, BAX partially counteracts BCL-2 [31]. It is possible that this is related to an unstable balance between proliferation and commitment of endometrial cells (see the “Altered autophagy” section).
6. CE enhances selectin E expression in endometrial microvascular endothelium. Selectin E is not normally found in microvessels of non-pathological endometrium. In particular, selectin E expression is stimulated by IL1b, TNF-a, and lipopolysaccharide [24]. However, selectin E has a clarified role in trophoblast migration within decidual spiral arterioles. For this reason, we can infer that a correct balance between selectin E and other selectins (L or P) is necessary for successful implantation, and an excessive expression of selectin E leads to inappropriate trophoblast invasion [36].
7. CE enhances CXCL13 expression in endometrial microvascular endothelium, when it is normally found only on endometrium surface. CXCL13 expression is only enhanced by lipopolysaccharide. It is likely that CXCL13 plays a role in selective recruitment of circulating naive B cells into the endometrium and has a key role on sustaining inflammation [24].
8. CE enhances CXCL1 expression in endometrial glandular epithelium, when it is usually not detectable and confined in the stroma. CXCL1 expression is enhanced by lipopolysaccharide [24] and, as above, it can have a key role in attracting stromal B cells into glandular epithelium [37].
9. In CE endometrium, there is an increase in IL-17 expression and a decrease in IL-10 and TGF-β1 expressions than in non-CE endometrium [38]. IL-10 and TGF-β1, in particular, are anti-inflammatory modulators and are secreted by Tregs. For this reason, they are both markers and mediators of a decreased inflammatory suppression and uNK cell recruitment [39].

Moreover, there is growing interest regarding the importance of mi-RNA regulation in CE. Recently, mi-RNA composition of exosomes has been studied in cows. In particular, exosomes are intercellular communication medium released by the endometrial epithelium into the uterine cavity and are involved in the transfer of signaling proteins (miRNAs and mRNAs) to either the embryo or adjacent endometrium. A difference between CE and non-CE endometrium has been identified, which requires further investigation. In particular, 118 miRNAs were found differentially expressed in the exosomes of cows without and those with endometritis. In those with CE, 52 miRNAs were found downregulated and 66 upregulated [40].

Women with CE have an altered endometrial expression in genes involved in inflammatory, cell proliferation, and apoptosis processes. CE is associated with shifted cytokine milieu toward Th17 over Treg immunity in endometrium. These findings support the notion that CE is associated with increased pro-inflammatory immune responses, which is often related to poor pregnancy outcomes [38]. CE upregulates both BCL-2 and BAX genes. As apoptosis is an important physiological mechanism in maintaining homeostasis during the menstrual cycle and in early phases of pregnancy, the altered anti- to pro-apoptotic factors ratio in women with CE could affect pregnancy-mediated tissue remodeling during blastocyst implantation and placental development in the uterus. Moreover, the prevalence of anti-apoptotic effect may explain the abnormal endometrial proliferation that is observed in women with CE, with the formation of endometrial micropolyps and, possibly, macropolyps [31]. Moreover, chronic inflammation may even predispose to endometrial carcinogenesis [41], even if this speculation needs robust scientific confirmation. CE is associated with the local release of different pro-inflammatory and oncogenic mediators such as nitric oxide (NO), cytokines (IL-1β, IL-2, IL-6, and TNF-α), growth factor, and chemokines. These mediators may make the endometrial inflammatory microenvironment more vulnerable toward tumorigenesis [42].

### Leukocyte infiltration

After cytokine hyper-expression, a new microenvironment is created in the endometrium, whose primary aim is no longer implantation, but immune defense against an exogenous agent. In a normal endometrium, B cells can be found in endometrial stroma, but not in surface epithelium, glandular epithelium, or glandular lumina. In CE, B cells are recruited in the functional layer and single cells can be found between epithelial cells and within gland lumina. On the other hand, T cells, NK cells, macrophages, and neutrophils do not show a different pattern compared with non-pathological endometrium [24, 43, 44]. For this reason, it can be assumed that CE inflammation relies on a B cell response. More specifically, CE is characterized by a specific Ig-class expression. CE endometrium has a higher density of Ig-bearing endometrial stromal cells than in physiological endometrium. The density of IgM+, IgA1+, IgA2+, IgG1+, and IgG2+ is higher, but IgG2+ is the most predominant and peculiar Ig-class in CE [45]. In particular, IgG2 is the main effector against bacterial capsular poly-saccharide antigens [46]. For this reason, this unique Ig subclass expression in CE is likely to result from in situ production of endometrial plasmacyte infiltrates as a response to infectious trigger [45].

Over the last 30 years, there has been growing interest in a peculiar class of leukocytes in relation to embryo implantation, namely the uNK. These cells have been detected in areas of stromal decidualization, including progesterone-treated endometrium, intrauterine decidua in ectopic pregnancy, and extrauterine decidua in normal pregnancy. They probably play a role in correct decidualization by promoting adequate vascular remodeling [47, 48]. In CE, during the endometrial secretory phase, the endometrium showed a lower percentage of uterine NK cell (CD56+ CD16- and CD56^bright^CD16-) [49]. This finding may suggest that a different leukocyte pattern may affect endometrial decidualization in CE and, subsequently, embryo implantation. Furthermore, in order to clarify the immunomodulation role of uNK cells, we considered that uNK cells, but not peripheral blood NK cells, were found to selectively express the genes of immunomodulatory proteins. In particular, they express glycodelin and galectin-1, tetraspanins, integrins, lectin-like receptors, and inhibitory receptors in early pregnancy (KIRs) providing a locally immunosuppressive environment at the maternal-fetal interface [50–53].

Thus, uNK are probably involved in both proper endometrial vascularization and immunomodulation. On the contrary, in CE endometrium, a decrease in these cells is observed which may probably impair implantation.

With regard to T lymphocytes, Matteo et al. found a higher percentage of CD3+ cells in CE endometrium and, in particular, the percentage of CD4 cells resulted significantly higher [49]. Interestingly, previous studies have confirmed the important role of Treg cells in regulating local immunomodulation during the implantation window. The semi-allogenic embryo transplantation is tolerated by a peculiar balance of Th1/Th2 and Th17/Treg cell immune responses [54]. In particular, Tregs suppress maternal alloreactive immune responses against paternal antigens in trophoblast thanks to the production of TGF-β and IL-10, anti-inflammatory mediators. Moreover, among the CD4+ T cells, approximately 10 to 30% express the Treg transcription factor FOXP3. A complex interaction between uNK and Tregs and proper vascularization and decidualization are under study. For this reason, an unstable balance of T cells can affect the acceptation of the embryo by the endometrium [39].

### Altered uterine contractility

Under normal conditions, a varied wave contractility pattern is present in the endometrium for the duration of the menstrual cycle. From an academic perspective, the contractility waves can be divided into three patterns: fundus to cervix (anterograde), cervix to fundus (retrograde), opposing (conflicting waves starting simultaneously on the fundus and the cervix and meeting in the middle of the uterus), not propagating (myometrial activity starting chaotically from different sites), and absent. In addition, cycle-dependent changes can also be observed in the characteristics of endometrial wave (EW) patterns. More specifically, during the early follicular phase anterograde, EWs predominate and are characterized by high amplitude and frequency. For the duration of the periovulatory phase, EWs are, for the most part, retrograde. EW activity is, to a large extent, absent throughout the secretory phase. From a physiologic and clinical perspective, it has been speculated that the purpose of anterograde EW is to empty the uterine cavity during the menstrual and early proliferation phases. It may be that retrograde EW during the periovulatory phase is connected with the active transport of sperm from the vagina to the fallopian tubes, while the quiescent status in the luteal phase may facilitate the process of embryo implantation [55–58].

Pinto et al. in 2015 studied the uterine contractility in non-pregnant women with CE and demonstrated that cycle-dependent uterine contractility is altered in women affected by CE [59]. In particular:

1. CE EW showed a 3.3 times lower occurrence of retrograde contractions during the periovulatory phase compared with non-CE uterus.
2. CE EW in midluteal phase showed a propagating contractile activity that was either anterograde or retrograde in 20% of cases. By contrast, non-CE EWs were local and non-propagating.

The EWs originate from the junctional zone (JZ), which is the transitional interface between the endometrium and the outer myometrium [60]. Different endometrial and myometrial pathologies can potentially cause alterations of the JZ thickness and activity, such as adenomyosis and fibroids [61]. For this reason, Pinto speculated that even the chronic inflammatory process, which occurs in chronic endometritis, could affect JZ functionality. In fact, the altered leukocyte population and the altered pattern of paracrine factors could influence the nearby endometrium of the JZ, inducing an altered contractility which can lead to infertility.

### Altered vascularization

Blood vessels are important players in the inflammatory process. Moreover, they are the principal morphological element of functional polyps, where large caliber arterialized vessels are seen in the functional layer. For this reason, a new interest has grown in detecting relationships between polyps and CE. Starting with this consideration, we considered the following study.

Alterations in blood vessels in CE secretive endometrium were studied by Carvalho (2013) [62]. The chief anatomopathological modifications in CE are, in descending order:

1. High vascular density with endothelial proliferation and swelling associated with hyaline vascular wall thickening with luminal occlusion
2. Hyaline thickening of the vascular wall with luminal occlusion
3. High vascular density with endothelial proliferation and swelling
4. High vascular density with endothelial proliferation and swelling associated with small vessel thrombosis
5. Hyaline vascular wall thickening with luminal occlusion and small vessel thrombosis
6. High vascular density with endothelial proliferation and swelling associated with hyaline vascular wall thickening with luminal occlusion and thrombosis of small vessels
7. High vascular density with endothelial proliferation and swelling associated with hyaline vascular wall thickening with luminal occlusion with luminal occlusion and segmented fibrinoid degeneration
8. Small-vessel thrombosis.

According to the study, in 85.7% of cases, vascular changes were associated with CE, while CE without vascular changes was observed in only 7.3% of cases. It was specifically noted that these vascular alterations are almost identical to the thick-walled vessels along the vascular axis of polyps. As a result of these observations, it can be hypothesized that the vessel axis of polyps may represent a developing stage of vasculopathy related to CE. Furthermore, the close relation between endometritis, polyps, and infertility is currently being studied [63]. The relationship between endometrial polyps and CE has recently been investigated, and one of the principal outcomes has been that EPs are often immunoreactive for CD-138, implying a possible role of chronic endometrial inflammation in their pathogenesis [64]. In all probability, these three diseases share a unique pathological substrate, and inflammation is the most likely candidate. However, further studies are needed to prove this relationship.

These pathological findings have the potential to impede endometrium receptivity, and subsequently implantation, in a range of different ways. Firstly, that increased vessel density is associated with recurrent early pregnancy loss must be taken into consideration. A possible reason for this may be found in the increase of environment oxygenation in the early phase of implantation, owing to the harmful effect of reactive oxygen species. By contrast, normal placental development takes place in a relatively hypoxic environment [48]. Secondly, flow reserve could be impaired by swelling, endothelial proliferation, small-vessel thrombosis, and luminal occlusion. This consideration could be comparable to microcirculation pathology in the myocardium. In this situation, when microvessels are affected by luminal stenosis, coronary flow reserve is reduced [65]. Analogously, it could be conjectured that what we can define as “endometrial flow reserve” can be affected by luminal impairment. Specifically, this blood perfusion could be reduced in instances where it is requested for placental development. To summarize, both excessive and reduced vascular efficiency may lead to a deleterious success for embryo implantation.

### Altered decidualization

Endometrial stromal cells (ESCs) participate in the process of decidualization. Estrogen and progesterone modulate decidua in order to facilitate embryo implantation and IGF-1 and IGF-2 are mediators for hormone actions [31]. In particular, estrogen stimulates IGF-I gene expression in the endometrium [66] and IGF-II is upregulated in decidualized endometrium in women treated with levonorgestrel [67]. However, more studies are needed to clarify the relationship between the IGF pathway and sexual hormones in decidualization.

At the beginning of this cascade, it is important to understand that the estrogen receptor (ER) expression reaches a peak in the early proliferative phase with a slight decrease in the late proliferative and early secretory phases in both glandular and stromal endometrium. It then shows a marked decrease in the late secretory phase, specifically in glandular cells. On the other hand, the progesterone receptor (PR) expression increases gradually in the proliferative and secretory phases and decreases in the late secretory phase, especially in the glandular cells [68].

This hormonal pattern is a leading promoter of decidualization. Specifically, decidua allows nutrition and gas and waste exchanges between the fetus and mother in cooperation with the placenta and also produces hormones, growth factors, and cytokines such as prolactin (PRL), corticotrophin-releasing factor (CRF), insulin-like growth factor binding protein-1 (IGFBP-1), and interleukin-15. Decidua probably also modulates trophoblast invasion and maternal immune system tolerance [69–71].

Two studies were considered in order to clarify the role of CE in endometrium decidualization: Wu (2017) and Mishra (2008), who both studied the impact of CE on decidualization [34, 72]. The main findings can be summarized as follows:

1. PRL and IGFBP-1 are markers of decidualization and are both decreased in CE [34]. Importantly, PRL level expression decrease has been previously correlated with spontaneous early pregnancy loss. In particular, PRL role in embryo implantation can be related to detrimental peptides inhibition (e.g., IL-2) and cytokine stimulation (e.g., TNF-a). Consequently, these cytokines can recruit immune cells, sensitize the ESCs to Fas-mediated apoptosis, and promote the release of molecules that interact with the blastocyst and allow a successful implantation [73]. By contrast, IGFBP-1 is part of a more complex pathway, the forkhead transcription factor, forkhead box O1A (FOXO1). In particular, IGFBP-1 plays a role in endometrial cell differentiation [74].
2. In patients suffering from both endometriosis and CE, the concentration of PRL and IGFBP-1 produced tend to be lower compared with that in patients with endometriosis without CE [34].
3. It is likely that the decrease in PRL and IGFBP-1 has its origin in gene expression. The mRNA levels of both, indeed, are lower in CE. As above, in this case, a similar tendency is also noted in patients with endometriosis [34].
4. In ESCs, expressions of ERα and ERβ (estrogen receptors α and β) and PRA and PRB (progesterone receptors A and B) are higher in CE endometrium than in non-CE endometrium [34, 72].
5. The cell number of decidualized ESCs is increased in CE decidua [34]. In particular, a clear cell proliferation can be noticed if considered Ki-67. Ki-67 is a marker of cell proliferation and its expression in the endometrial cells reaches a peak in the follicular phase and then decreases by half during the early luteal phase and drops during the mid- and late-luteal phase. Ki-67 in the endometrial glandular and stromal cells is significantly higher in both granulomatous and CE than the control group [72].

This data suggests that CE modifies decidualization. In particular, CE slows down decidua maturation by reducing estrogen and progesterone action. If the real cause is unknown, we can probably observe that the receptors are upregulated and speculate that inflammation may be responsible for sexual-steroid resistance. Previously, it was noticed that the loss of PR activity during early pregnancy resulted in the resorption of implantation sites [75]. Moreover, it has been proven that progesterone promotes ESC differentiation [76]. ERα and PRA are the predominant isoforms that mediate the critical functions of the uterus for the embryo implantation and maintenance of pregnancy [77, 78]. Taken together, these findings can help hypothesize that estrogen and progesterone action is decreased in CE endometrium and lead to altered embryo implantation.

To conclude, we can speculate that there are a less differentiation stimulus and a greater potential to proliferation in CE endometrium, which results in a greater number of ESCs with reduced commitment. As a result, the abnormal expression of sex hormone receptors linked to a greater ESCs number brings about the difficulty for the endometrium to prepare the field for a correct implantation.

### Altered autophagy

Autophagy is a mechanism of lysosome-mediated protein degradation that is necessary in maintaining cellular homeostasis by recycling amino acids, reducing the amount of damaged proteins, and modulating intracellular protein levels in response to extracellular signals. In particular, autophagy has important effects on the induction and modulation of the inflammatory reaction, modulates Treg cells, supports their lineage stability, and increases their life-expectancy [79].

Considering the fact that CE is associated with increased pro-inflammatory immune responses, it has been demonstrated that in CE endometrium, the level of microtubule-associated protein 1A/ 1B-light chain 3 (LC3-II protein) is significantly higher, while the level of mTORC1 is significantly lower than in controls [37].

Firstly, it must be considered that LC3-II is the transmembrane form of LC3, a soluble protein that is distributed ubiquitously in mammalian tissues. LC3 exists in two forms: a cytosolic form (LC3-I) and a lipid phosphatidylethanolamine-conjugated form (LC3-II) that is inserted into both inner and outer membranes of the autophagosome. Increased numbers of LC3-containing vesicles and increased LC3 flux indicate active autophagosome formation and clearance [80]. However, LC3 increase can be detrimental for cells and promote cell death which could possibly impair endometrial cells and lead to altered implantation [81]*.*

Conversely, mTOR is a regulator of cellular metabolism and has a crucial role in regulating cellular autophagy [82]. mTOR action relies on integrating signals from the environment to the nucleus for the regulation of cell metabolism, proliferation, survival, and autophagy. Indeed, mTOR action in the placenta is positively correlated with birth weight of the infant [83]. In particular, mTOR is an autophagy negative modulator and modifies inflammatory responses by modulating immunoproteasomal degradation [84]. Furthermore, mTOR has a key role in the post-implantation period and its reduction can lead to reduced feto-placental growth and increased resorption rate [85].

Our pathophysiological model speculates on the integrating role of both LC3 and mTOR in CE endometrium in implantation. In particular, autophagy is enhanced, and this creates an unstable balance between proteins (or modulators) which should be released, and proteins which should be recycled, leading to an altered environment which is less receptive for implantation. Finally, according to previous findings, autophagy could have an important effect on the induction of inflammatory reaction in CE and sequential changes in local cytokine milieu.

### Strength and limitations

This review includes all the studies about CE pathophysiology regarding altered implantation. However, most studies were primarily case-control, cohort studies, and retrospective. For this reason, various biases could arise from these study designs.

## Conclusion

**Fig. 1:**
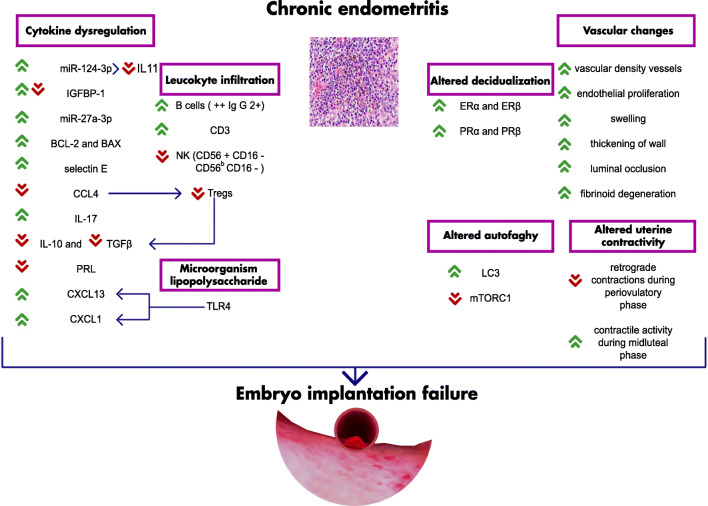
Chronic endometritis pathway

To the best of our knowledge, ours is the first summary of evidence on CE pathophysiology and its relationship with altered implantation. CE may affect female fertility in multiple ways, starting from an alteration of endometrial microbiota and continuing with inflammation and its secondary effects. Abnormal cytokines and leukocyte expression may impair the immune tolerance of the endometrium to the embryo and alter endometrial vascular permeability, potentially damaging embryo viability and trophoblast invasion. Additionally, abnormal uterine contractility during the midluteal phase may inhibit in vivo fertilization and affect transuterine migration of the embryo before implantation. Finally, altered autophagy may affect endometrial cell commitment and impair endometrial decidualization in women with CE. Figure 1 summarizes all the findings and helps visualizing the complexity of our model regarding the chronic endometritis pathway.

## Data Availability

All data are provided with this review.
